# Trajectories of quality of life and their association with anxiety in people with dementia in nursing homes: A 12-month follow-up study

**DOI:** 10.1371/journal.pone.0203773

**Published:** 2018-09-11

**Authors:** Alka R. Goyal, Sverre Bergh, Knut Engedal, Marit Kirkevold, Øyvind Kirkevold

**Affiliations:** 1 The Research Centre for Age Related Functional Decline and Diseases, Innlandet Hospital Trust, Ottestad, Norway; 2 Norwegian National Advisory Unit on Aging and Health, Vestfold Hospital Trust, Tønsberg, Norway; 3 Department of Nursing Science, Institute of Health and Society, University of Oslo, Oslo, Norway; 4 Norwegian University of Science and Technology (NTNU) in Gjøvik, Faculty of Health, Care and Nursing, Gjøvik, Norway; University of Antwerp, BELGIUM

## Abstract

**Objectives:**

This study aimed to identify possible groups of people with dementia (PWD) in nursing homes with different trajectories of proxy-rated quality of life (QoL), and to explore how anxiety (along with other characteristics) was associated with these trajectories of QoL of PWD in nursing homes at a 12-month follow-up.

**Methods:**

We included 298 PWD aged 65 years and older from 17 Norwegian nursing homes. The Norwegian version of the Rating Anxiety in Dementia scale (RAID-N) was used to assess anxiety, defined as RAID-N score ≥12; proxy-rated QoL was assessed by Quality of Life in Late-Stage Dementia (QUALID). The assessments were made at baseline and after a mean follow-up period of 350 days (SD 12.3). A growth mixture model identified two distinct trajectories of QUALID scores. Association between the QUALID score trajectories and demographic and clinical characteristics were analyzed with logistic regression models.

**Results:**

Trajectory group 1 (206 participants) had a lower proportion of participants with anxiety, and a more stable and better QoL, compared to trajectory group 2 (92 participants) at a 12-month follow-up. In a multivariate logistic regression analysis, more severe impairment in activities of daily living, along with presence of anxiety, depression, agitation, and use of antipsychotics at baseline, were associated with belonging to the trajectory group with proxy-rated poor QoL.

**Significances:**

This study reveals that anxiety is associated with proxy-rated poor QoL of PWD at a 12-month follow-up. Efforts should be made to identify anxiety among nursing home residents with dementia to initiate treatment.

## Introduction

Dementia disorders are usually progressive; irreversible; and characterized by impaired cognition, impaired functions in activities of daily living, and behavioral changes that are often disruptive for the person him/herself and for his/her family. So far, there is no cure for the various brain disorders causing dementia. As the person’s daily life function is affected, the person gradually becomes dependent on others. Dementia along with higher age, behavioral disturbances such as delusions and hallucinations, and increased functional impairment are some of the main factors leading to nursing home admission [1, 2]. In Norway, about 40% of people affected with dementia live in institutions, mainly in nursing homes [3]. This transition, and the inevitable changes in a person’s life due to dementia, may provoke negative reactions and apprehensions in the person with dementia, which may affect the person’s quality of life (QoL). Many cross-sectional studies have investigated factors associated with QoL in people with dementia (PWD) in long-term care facilities [4], but few longitudinal studies have investigated characteristics that could possibly affect the course of QoL of PWD in nursing homes [5–8].

A person’s QoL has multidimensional aspects; according to a definition by the World Health Organization, quality of life is usually based on the person’s subjective experience of own life, objective criteria in the context of culture, and the values of the society in which the person lives [9]. People with mild to moderate dementia can provide reliable perceptions of their own QoL [7, 10], whereas assessment of one’s own QoL can be difficult for people with severe dementia [5, 7, 11]. In nursing homes, most PWD have moderate to severe stages of dementia [12, 13].Various dementia-specific QoL assessment scales are available that are rated by the PWD and/or by the health care personnel/family caregivers [11, 14]. There is evidence indicating discrepancies between self and proxy-rated QoL of PWD. Proxy-rated QoL may be influenced by caregivers’ or family members’ own perspectives of QoL [15, 16]. However, in the nursing homes, most of the residents with dementia have more frequent and direct contacts with the nursing care staff during a day than their own family members. Thus, proxy-based instruments based on nursing care staff’s direct observations of a person’s behavior, emotions, and daily needs––such as the Quality of Life in Late-Stage Dementia (QUALID)—are considered appropriate to assess QoL in persons with moderate and severe stages of dementia living in nursing homes [17]. The knowledge that nursing homes’ health care personnel have about their residents with dementia should be used in assessing the QoL of PWD [15].

It is well known that anxiety and depression are highly prevalent among nursing home residents with dementia, and that these affect their self-rated QoL [4, 18–20], but the association between anxiety and depression and proxy-rated QoL of PWD has been ambiguous [4]. A systematic review indicates that dementia-specific depression scales, such as the Cornell Scale of Depression in Dementia (CSDD) [21], have been used in research studies on QoL of PWD [4, 7]. Only three studies have reported an association between anxiety and QoL in PWD using a dementia-specific anxiety scale, such as the Rating Anxiety in Dementia (RAID) scale [22] [5, 7, 23].

Hoe et al. [23] reported that both self-and proxy-rated high QoL of care home residents with dementia was correlated with low levels of anxiety symptoms. At a 20-week follow-up, the study by Hoe et al. [5] reported that self-rated increased QoL was associated with fewer baseline anxiety symptoms, whereas fewer baseline depressive symptoms were associated with proxy-rated increase in QoL. In a study by Selwood et al. [7], self-rated poor QoL was associated with more anxiety symptoms at one-year follow-up among PWD.

Knowledge is sparse about the role of anxiety and associated risk factors in proxy-rated QoL of PWD over time, especially when anxiety is assessed with a dementia-specific anxiety scale. More longitudinal studies of anxiety and related risk factors for proxy-rated QoL of PWD may add to the knowledge of factors that are important for QoL. This, in turn, can help health care personnel in nursing homes develop appropriate, proactive care interventions and management to enhance the QoL of PWD in nursing homes.

The main aim of this study was to identify possible groups of nursing home residents with dementia with different trajectories of QoL, and to explore how anxiety, along with other demographic and clinical characteristics, was associated with these trajectories of proxy-rated QoL of PWD in nursing homes at a 12-month follow-up.

## Methods

### Participants

A set of 21 nursing homes from two counties in south-eastern Norway were approached to recruit participants for the study. In total, 298 women and men with dementia aged 65 years or older from 17 nursing homes participated in the study. The dementia diagnoses were based on the research criteria of either the Diagnostic and Statistical Manual of Mental Disorders, Fifth Edition (DSM-V) (n = 101, the validation study group) [24, 25], the International Statistical Classification of Diseases and Related Health Problems, Tenth Revision (ICD-10) (n = 60) [26], or the International Classification of Primary Care-2 (ICPC-2) P70 (n = 137) [27], as documented in the medical records. Residents who were terminally ill, had schizophrenia or did not understand Norwegian well were excluded from the study. Participants were ethnic Norwegians or Scandinavians, except for one who had lived in Norway for many years. The recruitment process is described in detail elsewhere [18].

The participants were assessed at two points: a baseline assessment (N = 298) between August 2014 and November 2015, and a follow- up assessment (N = 205) between September 2015 and September 2016, with a mean interval of 350 days (SD 12.3). The attrition of 93 participants from baseline to follow-up (31.2%) was due to death. The participants who moved to other wards within the same nursing homes (n = 17) or to other nursing homes (n = 8) were also assessed at follow-up. The reasons for participants’ shifting to other wards or nursing homes included deterioration in physical condition (n = 10), deterioration of neuropsychiatric symptoms (NPS) (n = 9), and organizational changes in the nursing home (n = 6).

### Assessment scales

Anxiety was assessed by the Norwegian version of Rating Anxiety in Dementia scale (RAID-N). Shankar et al. [22] developed the RAID scale to assess anxiety symptoms in patients with all stages of dementia. It includes 20 items, of which 18 can be scored from zero (absent) to three (severe) or U (symptoms unable to measure); total scores range between zero and 54, and higher scores indicate more severe anxiety. Two items (phobias and panic attacks) are not included in the total score. RAID-N was validated for the PWD living in Norwegian nursing homes, and a cut-off score of ≥ 12 indicates a clinically significant generalized anxiety disorder (GAD) in patients with dementia [25]. The RAID-N score was based on clinical observations, information from the patient’s medical records, and a short interview with the patient him/herself about symptoms over the past two weeks. In the present study, anxiety as clinically significant was defined as RAID-N score ≥ 12.

QUALID is a proxy-based instrument assessing QoL in people with late stages of dementia. The scale is translated into Norwegian and has acceptable psychometric properties in assessing the QoL of PWD living in Norwegian nursing homes [28]. QUALID has 11 items that assess both positive and negative dimensions of behaviors considered to be indicative of QoL. Each item is rated from 1 to 5: the minimum score 11 indicates the best QoL, and the maximum score 55 indicates the poorest QoL. QUALID is administered as a structured interview of the caregiver, based on his or her direct observations of the person over the previous seven days [17]. QUALID is sensitive to assessing changes in QoL over a period of time, hence valid as a longitudinal measure of proxy-rated QoL [6, 29].

The participants’ dementia severity was assessed by the Clinical Dementia Rating scale, using sum of boxes (CDR-sob) [30]. Clinical Dementia Rating is a valid and reliable scale to categorize different stages of dementia [31]. CDR-sob correlates highly with the categorical score of Clinical Dementia Rating scale [30]. The CDR-sob rates six items individually, from zero to three (total CDR-sob scores zero to 18). Scores in the 4.5−9.0 range denote mild dementia, 9.5−15.5 moderate dementia, and 16.0−18.0 severe dementia [30].

Depressive symptoms were assessed by the Norwegian version of the CSDD [21, 32]. The CSDD is scored by using a short interview with the participant and a report from the primary caregiver based on direct observations. It has 19 items assessing depressive symptoms over the past week. The total score ranges between zero and 38, where a score ≥ 10 indicates clinically significant depression for nursing home patients [32]. In the present study, depression was defined as a score ≥ 10 on the CSDD.

Neuropsychiatric symptoms (NPS) were assessed by the Neuropsychiatric Inventory brief Questionnaire (NPI-Q) [33]. The ratings were based on the caregivers’ observations over the previous month. The questionnaire has 12 items assessing NPS severity, and the score ranges between zero and 36, with a higher score representing more severe NPS [33]. The 12 items of NPI-Q were grouped into four distinct sub-syndromes based on a factor analysis derived from the baseline study sample: psychosis (delusions and hallucinations), affective (dysphoria, anxiety, apathy, and appetite), agitation (agitation, irritability, disinhibition, and aberrant motor) and aroused (euphoria and nighttime disturbances) [18].

The participants’ activities of daily living (ADL) functions were assessed by the Physical Self-Maintenance scale (PSMS) [34], based on caregivers’ observations over the past week. The total score ranges between six and 30, with a higher score indicating more severe functional impairment. The participants’ general physical health was assessed by the General Medical Health Rating (GMHR) [35] based on participant’s present physical health status and regular medication use, and it included four categories: excellent, good, fair, and poor.

#### Assessment procedure

The assessment scales were administered by the participants’ primary nurses (in total, 57 nurses) or by the first author (ARG). In Norwegian nursing homes, the nursing care of the residents is organized under a primary nurse and a primary caregiver system. A primary nurse is a registered nurse (RN), who has the responsibility of a complete care plan of a resident under his/her care. A primary caregiver usually an auxiliary nurse, along with the primary nurse, has the responsibility to follow-up the care plan and other practical tasks of the resident. Before data collection, the primary and auxiliary nurses received 2–3 hours training from the first author in administering the assessment scales. The same nurse assessed the participant both at baseline and at follow-up. The same assessment scales were used at both assessment points. The primary nurses administered the assessment scales based on their direct observations of the resident, and a short interview with the resident when required. The first author interviewed the participants’ primary caregiver, and interviewed the participants when required. The first author was available for consultation during the data collection.

#### Information retrieved from the participants records

Demographic and clinical data—including age, gender, marital status, education, length of stay in nursing homes, dementia and other diagnoses, and participants’ regular use of medication—were collected by the participants’ primary nurses or by the first author.

Participants’ regular use of psychotropic medication were grouped into anxiolytics, antidepressants, antipsychotics, hypnotics and sedatives, and cognitive enhancers according to the Anatomical Therapeutic Chemical (ATC) index [36].

### Statistical analysis

Anxiety (RAID-N score ≥ 12) and depression (CSDD score ≥ 10) were dichotomized into present or absent. The GMHR scale was dichotomized into excellent/good and fair/poor. A growth mixture model was estimated to identify potential (unobserved) groups of participants following distinct trajectories of QUALID scores throughout the study period. Baseline differences in demographic and clinical variables between the trajectory groups were analyzed with *t*-test for continuous, and χ^2^ test for categorical, scales. To determine the participants’ characteristics and related risk factors pertaining to QUALID scores trajectories, a bivariate and a multivariate logistic regression was carried out with QoL group as dependent variable (0 = better QoL, 1 = poor QoL). Association between the participants’ characteristics measured at baseline and follow-up and a trend in QUALID scores was assessed by estimating a linear mixed model with random effects for residents. We included fixed effects for time, coded as before or after the characteristics. The same independent variables were used in the linear mixed model as in the logistic regression.

Due to high multicollinearity between RAID-N and CSDD scores (baseline r = 0.72; follow-up r = 0.74), these variables were not used in the regression models. At baseline, the overlap of dichotomized values of RAID-N and CSDD were 54 out of 118 participants (48 with only anxiety and 16 with only depression); therefore, we chose to use dichotomized variables of RAID-N and CSDD scores in the models. The variable NPI-Q affective was not included in the models, due to multicollinearity with both RAID-N dich. and CSDD-dich. Correlation between baseline CDR-sob and PSMS sum was r = 0.63. *P*-values < 0.05 were considered statistically significant. IBM SPSS Statistics, Version 23.0 (Armonk, NY, USA) was used for the descriptive statistics, for analyses of the differences within and between the groups, and for the logistic regression. MLwiN, Version 3.01 (Bristol, UK) was used for the linear mixed model.

### Ethical issues

The study was approved by the Regional Committee for Medical and Health Research Ethics (REK) in south-eastern Norway (ref. 2012/1958). Written informed consent was collected from the participants (n = 106) or from the next of kin if the participants did not have the capacity to give consent (n = 192).

## Results

At baseline, the participants’ (N = 298) mean age was 85.5 years (SD 6.8), and 73.2% were female. Participants who died during the follow-up (n = 93) were older [mean age 87.0 years (SD 5.5) vs. 84.9 years (SD 7.2), *p* = 0.005], had more impaired cognition [mean CDR-sob score 13.6 (SD 3.5) vs. 12.5 (SD 3.4), *p* = 0.012], and had more impaired ADL functions [mean PSMS- sum 17.4 (SD 4.4) vs.15.6 (SD 4.6), *p* = 0.001], but were not different in terms of severity of anxiety [mean RAID-N-sum 10.3 (SD 6.8) vs. 9.5 (SD 7.4), *p* = 0.367] or QoL [mean QUALID-sum 22.7 (SD 7.2) vs. 21.5 (SD 7.2), *p* = 0.174] than the participants who participated in the follow-up (n = 205).

**Fig 1:**
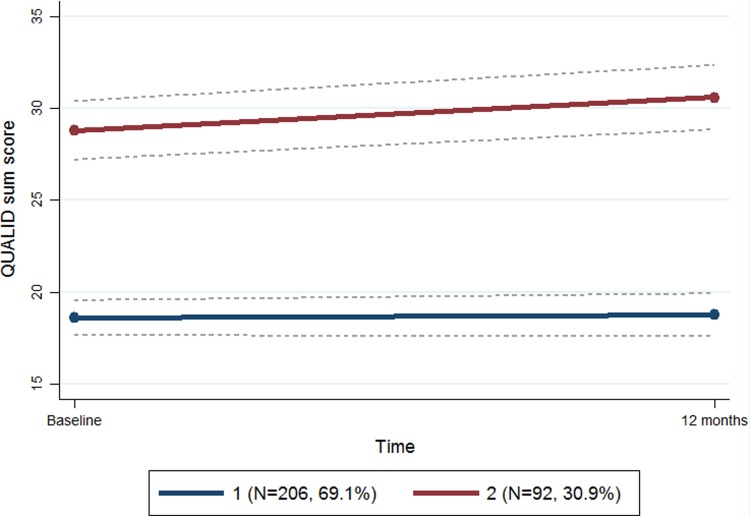
Trajectories analysis of quality of life by the quality of life in Late-Stage Dementia (QUALID).

The growth mixture model, based on QUALID scores, revealed two distinct trajectories of QUALID scores. Trajectory group 1 consisted of 206 participants and had a lower and more stable QUALID sum score (better QoL) compared to trajectory group 2. Trajectory group 2 consisted of 92 participants who had a higher and a non-significant increase in QUALID sum score (poorer QoL) during the study period (Fig 1). The average group probabilities were larger than 0.7, and 95% confidence intervals (CI) for the two trajectories did not overlap (Fig 1 and Table 1). Baseline characteristics of the two trajectory groups are presented in Table 2. Participants in trajectory group 2 (with poorer QoL) were more impaired in terms of cognition (higher CDR-sob score) and ADL functions (higher PSMS score), had poorer general physical health, had more anxiety and depression, had more severe NPS (higher NPI-Q score), and used more anxiolytics, antidepressants, and antipsychotics than the participants in trajectory group 1, see Table 2.

**Table 1:** Trajectories analysis of quality of life by the quality of life in Late-Stage Dementia (QUALID).

Variables	Trajectory group 1 (n = 206)		Trajectory group 2 (n = 92)	
	Regre. coeff. (SE)	*p*-value	Regre. coeff. (SE)	*p*-value
Intercept	18.59 (0.49)	< 0.001	28.80 (0.81)	< 0.001
Time	0.16 (0.66)	0.804	1.80 (1.04)	0.083
Average probability	0.92		0.86	

**Table 2:** Baseline differences between the trajectory groups. **Trajectory group 1 with better quality of life QoL, and trajectory group 2 with poorer QoL**.

Characteristics	Trajectory group 1 (n = 206)	Trajectory group 2 (n = 92)	*p*-value
Age in years, mean (SD)	86.0 (6.5)	84.6 (7.3)	0.102^1^
Female gender, n (%)	150 (72.8)	68 (73.9)	0.843^2^
Married, n (%)	49 (23.8)	16 (17.4)	0.217^2^
Education in years, mean (SD)	8.6 (2.3)^3^	9.1 (2.8)^4^	0.157^1^
Length of stay in days, mean (SD)	574.1(686.7)	524.3 (496.7)	0.532^1^
CDR-sob, mean (SD)	12.4 (3.4)	13.9 (3.3)	< 0.001^1^
PSMS-sum, mean (SD)	15.4 (4.5)	17.8 (4.5)	< 0.001^1^
GMHR-dich. (excellent/good), n (%)	96 (46.6)	26 (28.3)	0.003^2^
QUALID-sum mean (SD)	18.5 (4.6)	29.6 (5.9)	< 0.001^1^
RAID-N-sum, mean (SD)	8.0 (6.3)	13.9 (7.4)	< 0.001^1^
RAID-N dich., n (%)	47 (22.8)	55 (59.8)	< 0.001^2^
CSDD-sum, mean (SD)	5.0 (4.3)	10.0 (4.9)	< 0.001^1^
CSDD-dich, n (%)	24 (11.7)	46 (50)	< 0.001^2^
NPI-Q-sum, mean (SD)	4.9 (4.2)	10.6 (5.8)	< 0.001^1^
NPI-Q-psychosis-sum, mean (SD)	0.8 (1.3)	1.2 (1.6)	0.045^1^
NPI-Q-affective-sum, mean (SD)	1.4 (1.7)	4.1 (2.8)	< 0.001^1^
NPI-Q-agitation-sum, mean (SD)	2.1 (2.4)	4.1 (3.3)	< 0.001^1^
NPI-Q-aroused-sum, mean (SD)	0.6 (0.9)	1.2 (1.3)	< 0.001^1^
Anxiolytics, n (%)	21 (10.2)	24 (26.1)	< 0.001^2^
Antidepressants, n (%)	86 (41.7)	50 (54.3)	0.044^2^
Antipsychotics, n (%)	22 (10.7)	30 (32.6)	< 0.001^2^
Hypnotics and sedatives, n (%)	49 (23.8)	29 (31.5)	0.161^2^
Cognitive enhancers, n (%)	65 (31.6)	27 (29.3)	0.703^2^

^1^t- test;

^2^ χ^2^ test;

^3^n = 204;

^4^n = 90

CDR-sob, Clinical Dementia Rating Scale-sum of boxes; PSMS, Physical Self-Maintenance scale; GMHR-dich., General Medical Health Rating- fair /poor = 0, excellent/good = 1; QUALID, Quality of Life in Late-Stage Dementia; RAID-N, Rating Anxiety in Dementia-Norwegian version; RAID-N dich., RAID-N score <12 = 0, ≥12 = 1; CSDD, Cornell Scale for Depression in Dementia; CSDD-dich., CSDD score <10 = 0, ≥10 = 1; NPI-Q, Neuropsychiatric Inventory-Questionnaire; NPI-Q-psychosis (Delusions and Hallucinations); NPI-Q affective (Dysphoria, Anxiety, Apathy and Appetite); NPI-Q agitation (Agitation, Irritability, Disinhibition and Aberrant Motor); NPI-Q aroused (Euphoria and Nighttime Disturbances).

The adjusted multivariate logistic regression analysis revealed that a higher PSMS score (more severe ADL impairment), presence of significant anxiety and depression, higher score on the NPI-Q sub-syndrome agitation, and use of antipsychotics at baseline were significantly associated with belonging to trajectory group 2. Table 3 shows odds ratios of the various characteristics of the bivariate and multivariate analyses. The strongest association with belonging to trajectory group 2 was the use of antipsychotics, with an odds ratio of 4.53, followed by depression and anxiety, with odds ratios of 3.28 and 2.19, respectively. Between 29.7% (Cox and Snell R^2^) and 41.8% (Nagelkerke R^2^) of the variability was explained by the models.

**Table 3:** Logistic regression models predicting associating factors belonging to the trajectory group with poorer quality of Life (QoL) (n = 92). **Trajectory group with better QoL (n = 206) as reference**.

Variables	Bivariate models OR (95% CI)	*p*-value	Multivariate model OR (95% CI)	*p*-value
Age	0.97 (0.94; 1.01)	0.103	0.98 (0.94; 1.03)	0.472
Gender	0.95 (0.54; 1.65)	0.843	0.86 (0.40; 1.82)	0.685
Married	0.68 (0.36; 1.26)	0.219	0.67 (0.29; 1.53)	0.344
CDR-sob	1.15 (1.06; 1.25)	< 0.001	1.05 (0.93; 1.20)	0.436
PSMS-sum	1.13 (1.07; 1.20)	< 0.001	1.11(1.01; 1.21)	0.029
GMHR-dich	0.45 (0.27; 0.77)	0.003	0.76 (0.39; 1.48)	0.418
RAID-N dich	5.03(2.96; 8.53)	< 0.001	2.19 (1.10; 4.37)	0.026
CSDD-dich	7.58 (4.20; 13.68)	< 0.001	3.28 (1.50; 7.18)	0.003
NPI-Q-psychosis	1.20 (1.01; 1.42)	0.031	0.93 (0.75; 1.17)	0.554
NPI-Q-agitation	1.28 (1.17; 1.41)	< 0.001	1.15 (1.02; 1.30)	0.027
NPI-Q-aroused	1.63 (1.29; 2.04)	< 0.001	1.17 (0.87; 1.57)	0.306
Anxiolytics (no/yes)	3.11 (1.63; 5.95)	0.001	1.38 (0.61; 3.10)	0.438
Antidepressants (no/yes)	1.66 (1.01; 2.73)	0.044	1.10 (0.58; 2.08)	0.772
Antipsychotics (no/yes)	4.05 (2.18; 7.53)	< 0.001	4.53 (2.08; 9.87)	< 0.001

Trajectory groups, 0 = better QoL, 1 = poor QoL; Gender, 0 = female, 1 = male; Married,0 = unmarried/others, 1 = married; CDR-sob, Clinical Dementia Rating Scale-sum of boxes; PSMS, Physical Self-Maintenance scale; GMHR-dich., General Medical Health Rating- 0 = fair /poor, 1 = excellent/good; RAID-N dich., Norwegian version of Rating Anxiety in Dementia score,0 = <12, 1 = ≥ 12; CSDD-dich., Cornell Scale for Depression in Dementia score, 0 = <10, 1 = ≥10; NPI-Q-psychosis, Neuropsychiatric Inventory-Questionnaire (Delusions and Hallucinations); NPI-Q agitation (Agitation, Irritability, Disinhibition and Aberrant Motor); NPI-Q aroused (Euphoria and Nighttime Disturbances).

0 = no, 1 = yes.

The adjusted multivariate linear mixed model showed that higher PSMS score (β = 0.40, *p* <0.001); presence of significant anxiety (β = 2.23, *p* <0.001) and depression (β = 5.28, *p* <0.001); and higher scores on the NPI-Q sub-syndrome agitation (β = 0.46, *p* <0.001) were associated with increased QUALID score, representing a poorer QoL. On the other hand, presence of excellent/good general physical health was associated with a lower QUALID score, representing better QoL (β = -1.60, *p* = 0.003). An association between use of antipsychotics and increase in QUALID score did not reach a significant level (β = 1.33, *p* = 0.06) in the adjusted linear mixed model.

## Discussion

### Main findings

The present study revealed two distinct groups of PWD, with different trajectories of QoL: trajectory group 1 with better QoL, and trajectory group 2 with poorer QoL. In trajectory group 1 (which included approximately two-thirds of the participants), a lower proportion of participants had a RAID-N score of 12 and above (significant anxiety), and had more stable and better QoL compared with the participants in trajectory group 2. A RAID-N score of 12 and above was also associated with belonging to trajectory group 2 in the adjusted logistic regression analysis. The main findings associated with the trajectories of QoL will structure this discussion section.

Participants who died between the assessments were older and more impaired in terms of cognition and ADL functions than the participants who were alive at follow up; this is an expected finding. However, they did not differ in terms of anxiety or QoL from the rest of the participants. In line with previous findings, these results indicate that anxiety and QoL are not directly associated with mortality among PWD living in nursing homes [7, 37]. The QoL groups were different concerning several clinical variables, but in an adjusted logistic regression analysis, belonging to the trajectory group with the poorest QoL was associated with severity of impairment in ADL functions, presence of clinically significant anxiety and depression, high NPI-Q sub-syndrome agitation score, and use of antipsychotics at baseline.

In the present study, QoL in PWD was not independently affected by the severity of dementia itself, but we suggest that there is an indirect association between the factors—such as impaired ADL and severity of NPS, which are found to be related to the QoL of PWD [6–8]. The relationship between QoL, the severity of dementia and ADL functions is complex. A clear association exists between deterioration in cognitive impairment and ADL functions [38, 39]. However, in the present study, the correlation coefficient for the severity of dementia and ADL functions was <0.70, indicating that they can be treated as independent variables [40]. Furthermore, severity of dementia and impairment in ADL functions were both significantly associated with belonging to trajectory group 2 (with poor QoL) in the bivariate model; however, when controlling for each other, only ADL severity functions remained significantly correlated with belonging to trajectory group 2. Therefore, the consequence of dementia as expressed by impairment in ADL was a stronger factor associated with poor QoL than the severity of dementia itself.

A poor general physical health may also lead to decline in ADL function and to poor QoL in old PWD. Results of the linear mixed model of the present study indicate that participants’ excellent/good general physical health was associated with better QoL, a finding supported by previous studies [6, 12]. However, in our adjusted logistic regression analysis, poor general physical health was not significantly associated with belonging to the poor QoL trajectory group. The reason for the two analyses producing different results could be due to groupings. In the linear mixed model, we compared the participants’ general physical health with QoL both at baseline and follow-up; whereas, in the logistic regression model, the participants are dichotomized into two different trajectory groups, and the participants’ baseline GMHR scores are analyzed for association with belonging to one of the two trajectory groups. Different analytic strategies lead to different results, which indicate that the association between general physical health and QoL may be weak in PWD living in nursing homes.

Previous studies have reported that depression is highly associated with poor QoL in PWD [4]. Anxiety and depression often coexist in PWD, as in old people in general [18, 19, 41]. In the present study, anxiety controlled for depression was associated with belonging to the trajectory group with poor QoL, a result indicating that anxiety is an independent risk factor for poor QoL. This is an important finding of the study, due to two main reasons: scarcity of longitudinal studies on anxiety and QoL in PWD, and inconsistent findings of the association of anxiety with proxy-rated QoL. The results of the present study may contribute to reducing the ambiguity of the role anxiety plays in proxy-rated QoL of PWD over time [4], and may draw health care personnel’s attention to early detection and management of anxiety.

In the present study, all the NPI-Q sub-syndromes scores (psychosis, affective, agitation, and aroused) were significantly higher at baseline in the trajectory group with poorer QoL compared to the trajectory group with better QoL. The adjusted analysis indicated that participants with higher scores of NPI-Q sub-syndrome agitation are more likely to end up in the trajectory group with poorer QoL, compared to participants with lower NPI-Q sub-syndrome agitation scores. Many studies have reported that severe NPS, as assessed by caregivers, are associated with proxy-rated poor QoL in PWD [4, 6, 12]. NPS are prevalent in people in all stages of dementia [42]. A higher prevalence of NPS, especially agitation, imposes a challenge for PWD and a burden to the caregivers, thus reducing the QoL of PWD. Previous studies have also reported that more agitation is related to proxy-rated poor QoL [43–45]. A comprehensive assessment and management of agitation, anxiety and other NPS should be an important task for nursing home staff.

All else being equal, the use of antipsychotics inflicts the highest risk of ending up in trajectory group 2 (with poorer QoL), compared to participants not using antipsychotics. This is an another important finding of the present study, as only few studies have taken this factor into account concerning QoL of PWD, and previous findings were inconclusive [4]. A comparable study reported that the use of antipsychotics was related to proxy-rated poor QoL at baseline and increased mortality at a 10-month follow-up among PWD living in nursing homes [6]. Other studies have reported an association between the use of antipsychotics and increased mortality among PWD [46, 47]. NPS such as delusion, hallucination, and agitation among PWD are often treated with antipsychotics, even though the evidence for a positive effect is uncertain or limited [48, 49], with severe side-effects. To enhance the QoL of PWD, adequate management of agitation and other NPS by non-pharmacological interventions—such as TIME (Targeted Interdisciplinary Model for Evaluation and Treatment of NPS) and PCC (Person-centered care) based interventions may—play an important role [50–52]. It must be noted that the association between the use of antipsychotics and poorer QoL was not significant in the adjusted linear mixed model in the present study. However, the *p*-value of 0.06 shows a clear tendency of a relationship between the use of antipsychotics and QoL of PWD. This association needs further investigations, with utmost priority.

### Limitations and strengths

The QUALID scale is constructed to assess the QoL of people in late stage of dementia. To have a common QoL measure for all the participants of the present study, we chose the QUALID scale to assess QoL including people with mild to severe stages of dementia in the nursing home. This is in line with previous studies using QUALID scale for the assessment of QoL of PWD in the nursing homes and in geriatric psychiatric units [6, 51, 53]. Since QUALID is a proxy-rated scale, we may have lost valuable information on QoL perceived by people with mild to moderate dementia. The study was an observational study; it did not control for ongoing interventions or any other changes in the participants, such as comorbidities or shifting to other wards/nursing homes, which may have affected the results. Attrition can be a source of bias of main outcomes in follow-up studies (31.2% in present study), but here the attrition sample was not significantly different in terms of anxiety or QoL. The attrition sample was more impaired in ADL functions than the remaining participants, and this factor was taken into account by both regression models. The logistic regression included baseline variables of all the participants, whereas the linear mixed model included assessments at both time points.

The study population was nursing home residents with dementia, and the results cannot be generalized to other populations with dementia. A clear etiological dementia diagnosis, especially among the participants with unspecified dementia, may have added to the knowledge of QoL and anxiety in different dementia disorders. Many assessors involved in the assessment scales may have biased the results, although the assessors were trained in the administration of the scales and knew the participants well. With only two assessment points, the present study provides limited knowledge about the course of QoL of PWD in nursing homes. The strength of the study is its use of well-known, structured, and validated assessment scales; homogeneity of the study sample; and its prospective design.

## Conclusions

To our knowledge, this is the first study investigating association of anxiety with trajectories of QoL of PWD. Anxiety was found to be independently associated both with belonging to the trajectory group with poor QoL, as well as with proxy-rated poor QoL from baseline to 12-month follow-up. It is therefore important among nursing home personnel to increase awareness of and skills in identifying anxiety by validated dementia-specific anxiety instruments.
